# How digital technology enhances the efficiency and balances the value of primary healthcare services: a research in Tianjin, China

**DOI:** 10.3389/fpubh.2026.1841264

**Published:** 2026-07-15

**Authors:** Wei Wang, Xueqing Zhu

**Affiliations:** College of Public Administration and Law, Hunan Agricultural University, Changsha, China

**Keywords:** digital empowerment, precision supply, primary healthcare services, public health policy, service warmth

## Abstract

Against the backdrop of the deep integration of the Digital China initiative and the Rural Revitalization Strategy, the use of digital technology to empower primary healthcare services has become an inevitable trend. However, the paradox between precision and warmth needs to be resolved, and the scientific design and implementation of public health policies are key to addressing this challenge. Taking Tianjin's Primary Digital Health Community as a case study, this paper constructs a dual embedding analysis framework of “Process-Relational” to systematically explore the underlying mechanisms and practical pathways of digital technology empowerment, thereby providing theoretical support for public health policy formulation. The study finds that the core of digital empowerment lies in the synergistic interaction between process embedding and relational embedding: process embedding achieves precision across the entire service chain through standardized systems, integrated platforms, and AI algorithms, thereby establishing a foundation for enhanced efficiency; Relational embedding conveys human warmth by empowering frontline staff, connecting diverse stakeholders, and creating emotional spaces. Together, these form a virtuous cycle that drives primary healthcare services to achieve a triple leap in efficiency, governance, and value. The dual-embedding mechanism revealed in this paper offers practical insights for optimizing public health policies. It aids in formulating grassroots healthcare development policies that balance efficiency with equity, and technology with humanistic values, thereby promoting the high-quality development of grassroots public health services.

## 1 Introduction

The report of the 20th National Congress of the Communist Party of China outlined a grand blueprint for accelerating the development of a Digital China. Leveraging digital technologies to enhance the quality and efficiency of primary healthcare service delivery has now become a cornerstone of grassroots healthcare governance. The “Healthy China 2030” Planning Outline and the “National Health Informatization Plan for the 14th Five-Year Plan Period” emphasize deepening the development of “Internet Plus Healthcare (1),”

signifying that digital technology-driven primary healthcare service delivery has emerged as an effective pathway to lead and support the high-quality development of the health sector (2).

Digital technology is driving the implementation of precision-based, end-to-end healthcare services at the community level. By enabling accurate profiling of service demands, precise matching of service resources, precise control of service processes, and precise evaluation of service outcomes, digital empowerment holds the potential to resolve persistent issues in traditional primary healthcare, such as mismatches between supply and demand, resource wastage, and inefficiency, thereby achieving cost reduction, efficiency gains, and service optimization and upgrading (3). This represents the pursuit of instrumental rationality in service provision and is an inevitable requirement for enhancing government effectiveness. A paradox that cannot be ignored emerges. Does technological precision necessarily come at the expense of service warmth? In practice, over-reliance on automated processes may lead to impersonal and rigid service delivery (4). A unified online platform risks overlooking the personalized needs and varying digital literacy levels of vulnerable groups in grassroots communities, particularly the older adults and persons with disabilities, thereby creating new digital divides and emotional barriers. The human warmth inherent in public services, manifested through compassionate care, emotional interaction, trust-building relationships, and ethical values, now faces erosion by technological logic. This tendency to “prioritize technology over humanity” and “emphasize efficiency over fairness” contradicts the fundamental purpose of public services and risks sparking new social tensions.

Therefore, in the process of digitally empowering primary healthcare services, how can we achieve both precision and warmth? What are the underlying mechanisms at play? Currently, academic research on digital empowerment largely focuses on evaluating the effectiveness of technology applications (5), analyzing the causes of the digital divide (6), or introducing models in specific fields. However, few studies delve into the “technology-society” interaction mechanisms to systematically reveal the underlying logic of balancing precision and warmth. Given this, this paper aims to construct a Dual embedding analysis framework of “Process-Relational” to explore the question: Through what mechanisms can digital technologies achieve precise delivery of primary healthcare services while effectively conveying the necessary human touch? Using the innovative practice of Tianjin's Primary Digital Health Community as a case study, this paper dissects its underlying mechanisms to provide theoretical support and practical insights for resolving the “efficiency-warmth” paradox in current digital rural development initiatives. Help policymakers strike a balance between improving service enhances the Efficiency and Balances the Value of Primary Healthcare Services system.

## 2 Literature review

The dual tension between “precision” and “warmth” not only poses practical challenges but also forms the core debate in academic discussions about digital empowerment of public services. Existing research has largely crystallized into three perspectives. First is the technology-driven perspective, which emphasizes technological efficiency and offers an optimistic interpretation for achieving precision. Second is the socially critical perspective, which maintains high vigilance toward technological risks and profoundly reveals the crisis of human warmth erosion. Third is the technology-society systems perspective, which seeks to transcend the binary opposition and explore paths of integration. This chapter will systematically review these three perspectives, aiming to clarify their theoretical contributions and inherent limitations, thereby providing a research framework for this paper.

### 2.1 Technology-driven perspective

The technology-driven perspective tends to be optimistic, viewing digital technology as the “engine” driving transformation in the public sector. Early research focused on the role of e-government in enhancing administrative efficiency and increasing government transparency (7). With the advancement of technologies like big data and AI, the research focus has shifted toward smart governance, emphasizing that data-driven decision-making enables personalized, predictive, and proactive delivery of public services (8). In the realm of grassroots public services, scholars have explored how “Internet Plus Government Services” can overcome temporal and spatial constraints to achieve the goal of “letting data run more so people run less (9),” as well as the immense potential of digital technologies in areas such as targeted poverty alleviation, smart agriculture, and telemedicine (10). This perspective profoundly reveals the immense value of digital empowerment in precision dimensions. However, its potential pitfall lies in the risk of falling into technological determinism, with insufficient attention paid to the social, cultural, and ethical risks underlying technology application.

### 2.2 Social criticism perspective

In contrast to the technology-driven perspective, the social criticism perspective focuses on revealing the negative effects of digital empowerment. The core issue is the “digital divide,” which not only refers to the first gap in unequal access to devices and networks but also points to the second gap in digital skills and the third gap in the disparity of abilities to leverage digital technologies for socioeconomic benefits (11). This issue is particularly acute at the grassroots level, where groups such as the older adults and those with low educational attainment are prone to marginalization. Furthermore, Foucault scholars argue that digital platforms, as a new form of governance technology, may function as a “Digital Panopticon,” enhancing the state's capacity for surveillance and social discipline (12). The “black box” operation of algorithms may also entrench or even amplify societal biases, leading to algorithmic discrimination and undermining social equity (13). While this perspective is crucial for vigilance against technological risks and attention to human warmth and value dimensions, it sometimes overlooks the potential for actors to leverage technology for positive innovation and resistance against injustice, with limited research on constructive solutions.

### 2.3 Technology-society systems perspective

To transcend the above dichotomy, a third perspective has emerged. It no longer views technology and society as mutually independent or opposing entities, but rather as a “Technology-Society System” that mutually shapes and co-evolves (14). This perspective emphasizes that the ultimate impact of technology is not determined by the technology itself, but rather by how it is embedded within specific organizational structures, institutional rules, and interpersonal interactions. In the field of primary healthcare services, this implies that the value of digital tools lies in empowering people rather than replacing them. Concepts such as the “Street CEO” emphasize that technology should serve as an “enhancer” and “empowerment tool” for service providers (4, 15). Similarly, scholar Huang Xiaochun's research indicates that social governance innovations under Party-building leadership achieve effective coordination between vertical and horizontal forces through technological platforms (16). This perspective offers crucial insights for this study: the balance between precision and warmth must be found. While existing research has highlighted the synergistic relationship between technology and society, it has not yet fully elucidated the specific pathways through which digital empowerment mechanisms support public health policy making. There is a lack of targeted research that derives policy optimization directions by analyzing service practices in reverse, and this constitutes the core research gap addressed in this paper.

In summary, the technology-driven perspective provides theoretical justification for precision, yet overlooks the loss of warmth. The social criticism perspective keenly identifies the crisis of warmth, yet overlooks pathways to achieving precision. While the techno-social systems perspective points toward integration, its concept of embeddedness remains vague, failing to distinguish between process embedding and relational embedding and their underlying rational logic. Consequently, it cannot construct a complete causal chain explaining how precision and warmth can synergistic symbiosis. Therefore, inspired by the techno-social systems perspective, this paper attempts to bridge this gap. By analyzing the mechanisms through which digital platforms embed themselves into both process and relational dimensions, it systematically elucidates the intrinsic logic linking efficiency enhancement and value equilibrium.

## 3 Methods

### 3.1 Study design

This study adopts a single case study method. Case study is suitable for exploring in-depth mechanisms such as how things operate and why they work. Accordingly, this study focuses on the internal mechanism and value balance of digital empowerment in primary healthcare services, with a focus on analyzing the action mechanism of the process-relational dual embedding. This paper employs a single-case study design, drawing on publicly available secondary sources such as municipal policy documents, operational reports from the health and wellness system, and operational records of the Digital Health Community platform. Based on these texts, this study employs a secondary text inductive analysis method: centered on the core research question of how digital primary healthcare balances precision and warmth values, the study categorizes and organizes official materials from different levels and time periods, conducts cross-comparisons, and derives the objective practical characteristics formed by the implementation of Tianjin's Primary Digital Health Community. All theoretical conclusions are grounded in the practical facts recorded in the texts, ensuring the traceability of the analytical process.

### 3.2 Case selection and data sources

This study takes the Tianjin's Primary Digital Health Community as its case study. Launched in 2020, the initiative covers 266 primary-level healthcare institutions across the city and has established four major platforms: “Cloud Management,” “Cloud Pharmacy,” “Cloud Diagnosis,” and “Cloud Services.” As a prime example of digital transformation in China's primary-level healthcare sector, it features mature practices, accessible data, and measurable outcomes, making it well-suited for an in-depth analysis of the “precision-warmth” dual-embedded mechanism. The successful implementation of this case not only provides a typical example for analyzing the dual-embedded mechanism, but its lessons can also serve as a direct reference for similar regions in formulating public health policies related to the digitization of primary healthcare.

The data for this study were derived from publicly available secondary sources, including: Policy documents, implementation plans, and operational guidelines related to primary-care digital health consortia in Tianjin; Official operational reports and work summaries issued by health authorities; Public reports on the implementation and operational records of the “Cloud Management, Cloud Pharmacy, Cloud Diagnosis, and Cloud Services” platform; Materials such as summaries of practical experiences and situation reports from frontline medical staff and administrators. To ensure the rigor and credibility of the study, this research relies exclusively on publicly available authoritative sources; all interpretations adhere to the principles of logical consistency and evidence-based reasoning, avoiding subjective inferences to ensure an objective and standardized analytical process. To mitigate the institutional biases inherent in relying on a single official text, this study integrates four types of data policy documents, official operational reports, platform operation records, and frontline news reports and conducts cross-referencing. Only content corroborated by multiple sources is included in the analysis. At the same time, the study proactively identifies negative information in the documents. Such as shortcomings in rectification efforts and public grievances and conducts a dialectical interpretation to reduce the one-sidedness of narratives caused by relying solely on promotional texts.

### 3.3 Theoretical foundations

Through the synthesis and analysis of the data, it was found that the implementation of the Tianjin's Primary Digital Health Community has taken two parallel practical paths. The first involves establishing a unified digital platform and implementing standardized protocols for the entire diagnostic, treatment, and management process, with the core objective of reducing process costs and improving overall operational efficiency. The second involves optimizing the workload of primary-care medical staff and establishing long-term communication channels between doctors and patients for chronic diseases, thereby continuously maintaining trust and interpersonal service relationships between doctors and patients. These two types of practices form the basis for the empirical analysis in this study. Building on this foundation, this paper introduces Max Weber's dichotomy of instrumental rationality and value rationality as an analytical lens to provide a theoretical interpretation of the two practical pathways. The digital process transformation aimed at standardization and efficiency improvement aligns with the action logic of instrumental rationality. Which seeks to achieve predetermined goals through optimal means and thus gives rise to the concept of Process embedding; Conversely, the interactive service model centered on sustaining doctor-patient emotional bonds and upholding humanistic care corresponds to the action logic of value rationality, which pursues intrinsic values; from this, the concept of relational embedding is derived. In summary, the corresponding relational between instrumental rationality and process embedding, as well as between value rationality and relational embedding, is an empirical conclusion drawn from the analysis of local digital practice texts. Weber's relevant theories serve merely as analytical tools for interpreting real-world data and do not constitute *a priori* assumptions predetermined by the study.

Max Weber's dichotomy of rationality namely, instrumental rationality and value rationality offers a classic perspective for analyzing the inherent tension between “precision and warmth” in digital healthcare (17). Instrumental rationality is a logic of action guided by utility and efficiency. Its core lies in achieving predetermined goals through optimized, calculable means, while the legitimacy of the goals themselves is suspended (18). In this study, instrumental rationality manifests as the pursuit of “precision” in primary healthcare services, corresponding to the standardization, efficiency, and precision of service processes; value rationality refers to actors acting out of a conscious commitment to certain intrinsic values, the absence of which would deprive goal-oriented behavior of the sustained spiritual motivation needed to persevere (19). In this study, value rationality is the source of “warmth,” corresponding to the humanistic care, fairness, justice, and emotional warmth inherent in healthcare services.

Using Weber's dichotomy of rationality as the core analytical lens, combined with cutting-edge perspectives on digital governance in recent years, can further enrich the dimensions of the discussion. A series of existing studies have proposed a systemic transformation approach involving the coordinated transformation of technology, society, and governance; they critique development models that prioritize technology alone and advocate for a people-centered paradigm of digital public governance (20), while also emphasizing that, during the implementation of artificial intelligence, attention must be paid to the rights of vulnerable groups and mechanisms must be established to prevent fairness risks such as algorithmic discrimination. The conclusions of the aforementioned studies serve as supporting evidence for this research, further illustrating that relying solely on process optimization under instrumental rationality is insufficient to resolve the equity conflicts arising from digital transformation; it is essential to simultaneously establish a balancing mechanism grounded in humanistic services guided by value rationality.

Based on the above cutting-edge research perspectives, the high-quality development of digital healthcare requires shifting public services from a technology-driven paradigm of instrumental rationality to a governance paradigm that prioritizes humanistic values. This involves establishing a dynamic balance between improving the efficiency of digitalized processes and preserving relational humanistic values, ultimately achieving a symbiotic unity of precision and warmth. Therefore, within Weber's theoretical framework, the core issue of this paper is more profoundly reframed as follows: in a process of digital empowerment dominated by the logic of instrumental rationality, how can actors proactively harness technology to resist its comprehensive erosion of value rationality, thereby identifying viable pathways for the survival and even flourishing of service warmth?

### 3.4 Analytical framework

In today's era where digital technology is sweeping through public services, the paradox of “precision and warmth” has emerged as a pervasive governance challenge. This paradox is not a novel phenomenon, but rather a contemporary manifestation of the core tensions inherent in modern society. To uncover its underlying mechanisms, this study will ground its theoretical foundation in Max Weber's seminal theories on social action and the fate of modernity. Building upon this foundation, we will construct an operational “Process-Relational” Dual embedding analytical framework.

**Figure 1:**
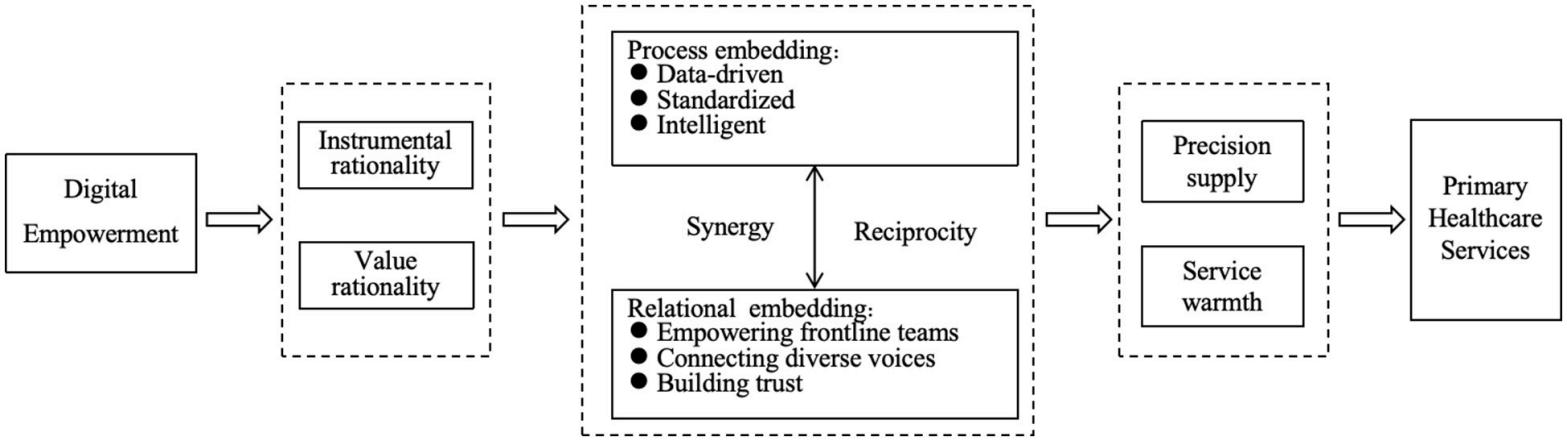
Dual embedding analysis framework of “Process-Relational”.

Digital platforms themselves are neutral, their value effects depend on the depth and dimensions of their embeddedness. Only when a platform achieves deep integration into both service processes and social relationship networks can it synergistically deliver precise efficiency gains and warm-hearted value transmission. Therefore, to systematically explain how digital empowerment achieves both precision and warmth, this paper constructs a dual embedding analysis framework of “process-relational” (see Figure 1) to address the contemporary need for primary healthcare to shift from an efficiency-oriented approach to a people-centered one.

#### 3.4.1 Process embedding: enabling precision supply

Process embedding refers to the deep integration of digital technologies into the entire workflow of primary healthcare services, including service identification, resource allocation, service delivery, and performance evaluation. By standardizing, digitizing, and intelligentizing these processes, it enables precise service provision, thereby addressing the dimension of technological efficacy (21). At its core, this approach reshapes the service delivery system through instrumental rationality, forming the fundamental basis for enhancing efficacy.

Process embedding centers on the core dimensions of technological standardization, resource consolidation, and service intelligence. Specifically, this manifests as the unified standardization of service processes, the platform-based coordination of data and resources, and the intelligent support provided by AI technology in areas such as diagnosis and treatment, review, and follow-up. The underlying mechanism is to enhance service efficiency, reduce operational costs, and improve the precision of service delivery through the integration of digital technologies. Through the use of unified platforms, resource sharing, AI-assisted diagnosis, and intelligent prescription review, process embedding enables precise operations across four levels: demand identification, resource allocation, process control, and performance evaluation, thereby providing a solid foundation for service quality improvement. However, it is important to note that isolated or excessive process embedding may lead to algorithmic bias, operational rigidity, and the digital divide key factors contributing to a lack of warmth in service delivery (22).

#### 3.4.2 Relational embedding: conveying service warmth

Process embedding addresses efficiency issues, while relational embedding addresses value issues; the two complement each other (23). Relational embedding refers to integrating digital platforms into the social structures and interpersonal networks of communities. By empowering frontline service providers, facilitating communication and collaboration among diverse stakeholders, and building social trust, it transforms technological platforms into vehicles for conveying humanistic care and fostering social cohesion. At its core, this approach guides technological application with value-oriented rationality, upholds the centrality of the human subject, and uses regulatory frameworks to prevent the excessive expansion of instrumental rationality serving as the key to ensuring the human touch in service delivery (24). Its implementation mechanism lies in transforming technology from a surveillance tool for “street-level bureaucrats” into an empowerment tool, thereby connecting diverse stakeholders and solidifying the foundation of trust (25).

Relational embedding integration centers on trust between doctors and patients, multi-stakeholder collaboration, and humanistic care, and is manifested in three specific ways: first, freeing healthcare workers from administrative tasks to increase time for face-to-face communication; second, promoting the coordination of service networks across different entities and departments; and third, providing residents with health companionship, emotional support, and patient responses. The causal mechanism lies in building relationships to enhance residents' trust, improve service experiences, and ensure equitable access for vulnerable groups. The ultimate outcomes are reflected in three dimensions of “warmth”: the humanization of services, community cohesion, and government legitimacy. At its core, this approach empowers frontline healthcare workers, connects diverse services, and builds spaces for trust and emotional connection, thereby preventing technology from replacing humanistic care. Without effective process embedding to support it, relational-based embedding alone can lead to non-standardized and fragmented service delivery, which in turn reduces overall operational efficiency.

#### 3.4.3 Synergy and reciprocity: the path to dual embedding

The ability to achieve both “precision” and “warmth” stems from the synergy and mutual reinforcement between process embedding and relational embedding. These two elements do not operate independently but are organically combined within a unified platform, creating a virtuous cycle. Process embedding lays the groundwork for relational embedding. The automation and standardization of processes free frontline healthcare workers from a large volume of repetitive and tedious administrative tasks, allowing them to devote more time and energy to relationship-building activities such as communicating with residents and providing emotional support. Relational embedding optimizes and reinforces process embedding. The trust and interaction established through relational embedding make residents more willing to provide authentic and comprehensive personal information, thereby improving data quality and reliability, which in turn enhances the precision of process embedding. At the same time, based on their in-depth understanding of residents, healthcare providers can identify flaws in automated processes and algorithmic blind spots, and propose optimization suggestions, making the technical system more adaptable and user-friendly. The synergy between these two elements drives primary healthcare to shift from being technology-driven to being driven by human-centered values, achieving a harmonious balance between precision and warmth.

## 4 Context

The Tianjin's Primary Digital Health Community, launched in 2020, covers 266 primary healthcare institutions across the city. By establishing four cloud platforms “Cloud Management, Cloud Pharmacy, Cloud Diagnostics, and Cloud Services”, it has systematically enhanced primary healthcare service capabilities. Its success lies precisely in achieving dual integration of the digital platform into both service processes and doctor-patient relationships.

### 4.1 Process embedding: building the technical foundation for precision medicine

Through a series of digital transformations, Tianjin's primary Digital Health Community has achieved precise management across the entire medical service process, establishing a model practice of process integration.

#### 4.1.1 Replace ambiguous management with standardized systems to achieve operational precision

Tianjin's primary Digital Health Community has uniformly adopted municipal-level cloud hospital information systems (HIS) and cloud public health systems. These systems replace the previous management and accounting models at community health service centers which relied on manual experience and inconsistent standards with standardized data interfaces and business logic. Key processes such as medication inventory, financial receipts and expenditures, and performance accounting have been automated and made real-time, significantly enhancing management efficiency and transparency while laying the data foundation for subsequent precision incentives.

#### 4.1.2 Replace fragmented resources with an integrated platform to achieve precise matching

Addressing pain points such as expensive yet underutilized grassroots examination equipment and incomplete drug inventories, the “Cloud Diagnostics” and “Cloud Pharmacy” platforms play a pivotal role. The “Cloud Diagnostics” platform establishes regional sharing centers to centrally manage resources including 635 networked devices and 41 cloud-based mobile examination kits, replacing the fragmented, independent configurations previously adopted by individual institutions. Residents can now undergo examinations at nearby facilities, with results transmitted to senior specialists for diagnosis via the cloud, enabling precise matching and decentralization of high-quality diagnostic resources. The “Cloud Pharmacy” platform integrates 1,560 drug varieties and 2,785 specifications, addressing the limited drug catalogs at primary-level institutions. Through an “online prescription processing + offline logistics delivery” model, it precisely meets patients' medication needs.

#### 4.1.3 Replacing human judgment with AI algorithms to achieve precise service delivery

The healthcare consortium leverages artificial intelligence to enhance service precision. The “AI Doctor” intelligent diagnostic assistance system analyzes vast medical datasets to provide general practitioners with diagnostic recommendations and risk alerts, improving treatment plan quality by 22%. The “AI Smart Prescription Review” system replaces pharmacists' repetitive preliminary review tasks, raising prescription compliance rates to over 98%. These practices exemplify Process embedding intelligence, effectively reducing human error and elevating service professionalism.

### 4.2 Relational embedding: a humanistic bond between doctors and patients

Relational embedding transforms the Digital Health Community into not only an efficient machine but also a service network imbued with humanistic care.

#### 4.2.1 Empowering family doctors to rebuild trust between doctors and patients

The Digital Health Community Platform is not intended to replace family doctors but to serve as a super toolkit for their community service. Through the cloud-based platform and mobile terminals, family doctors can access contracted residents' health records and follow-up logs in real time, conduct online consultations, and provide health guidance. This significantly enhances the frequency and depth of doctor-patient interactions. More importantly, the platform liberates family doctors from cumbersome paperwork, allowing them to dedicate more time to in-person emotional exchanges such as home visits and health education.

#### 4.2.2 Connecting diverse stakeholders to build a collaborative service network

The “Cloud Services” platform not only connects doctors and patients but also links broader social service resources. It centrally handles “Internet+nursing” requests, providing in-home services for older adults individuals who are incapacitated or partially incapacitated. By 2024, the service had reached 226,000 individuals. This achievement stems from the platform's ability to organically coordinate professional nurses, volunteers, community workers, and other stakeholders into a collaborative service network centered on seniors' needs. This multi-to-one care model far exceeds what any single doctor could provide, embodying warmth and compassion.

#### 4.2.3 Patient-centered, creating spaces for emotional connection

The Digital Health Community has established 238 chronic disease management centers, providing continuous health management and peer support services to nearly 230,000 patients through WeChat groups, mobile apps, and other channels. These online communities serve not only as channels for disseminating health knowledge but also as spaces for emotional support where patients encourage one another and share experiences with health managers. This bond forged through shared experiences effectively alleviates patient anxiety and enhances treatment adherence, vividly demonstrating how digital platforms can build “representational spaces” and convey humanistic care. Of course, online communities only reach people with basic digital literacy. For older adults individuals living alone who lack any smart devices and have severe disabilities, online channels leave service gaps; local areas rely on in-person home visits to fill these gaps. This is a supporting measure that must not be overlooked when replicating this model in other regions.

While online digital processes and online community services enhance efficiency and strengthen the doctor-patient relationship, data collection, AI algorithms, and online service models give rise to a series of ethical risks in digital healthcare. Drawing on the practices of the Tianjin Healthcare Consortium, this article analyzes the underlying ethical constraints from five key dimensions. Throughout the process of digitally empowering primary care, data collection, algorithm application, and online services give rise to multiple ethical risks. Relying solely on digital platforms makes it difficult to ensure that technology serves the greater good; therefore, a comprehensive and supporting governance system must be established. First, personal data security governance. The city's “Four Clouds” system aggregates residents' complete health records and manages data risks through tiered accounts, audit trails, and encrypted storage. However, staff turnover at the grassroots level and the integration of third-party service providers still pose risks of data leaks, and a routine access control audit mechanism urgently needs to be improved. Second, health privacy and informed consent. The platform's authorization agreements are written in technical and obscure language, making them difficult for the older adults and those with lower levels of education to fully understand. Current authorizations distinguish between two scenarios immediate use for medical treatment and public health research. But there is a widespread practice of users selecting all options with a single click, rendering the informed consent process largely a formality. A more refined, tiered authorization mechanism is needed to safeguard patients' right to make autonomous choices. Third, algorithmic bias and algorithmic governance. AI-based diagnostic and prescription review models are trained using samples of common urban diseases, resulting in insufficient adaptability to rare geriatric diseases in rural areas and leading to disparities in equity across population groups. Although manual review mechanisms are in place, there is a lack of routine systems for assessing algorithmic fairness and iterating on training samples, and there are shortcomings in algorithmic explainability. Fourth, the boundaries of non-clinical data use. Residents' health data is used in non-therapeutic contexts such as resource allocation, epidemiological statistics, and academic research. Local regulations require that data be fully desensitization before it can be shared; however, current desensitization standards and interdepartmental approval processes are lax, which can easily lead to implicit privacy violations. Clear ethical red lines for data reuse must be established. Fifth, the trade-off between efficiency and individual rights. Process embedding enhances service efficiency through the aggregation of data across all domains, but it carries risks such as the excessive collection of personal health information and the monitoring of online behavior through comprehensive tracking. Relational embedding, on the other hand, incorporates services such as in-person service counters and home visits, which can reduce the mandatory online data collection from vulnerable groups, striking a balance between efficiency goals and citizens' privacy and right to choose their own healthcare providers.

In summary, digital tools cannot spontaneously resolve ethical conflicts such as privacy and algorithmic fairness. It is essential to uphold the human-centered principles of digital healthcare. The ethical risks mentioned above cannot be addressed solely through the digitization of processes. Instead, the Dual embedding analysis framework of “Process-Relational” in this paper relies on human-centered services, such as in-person service counters and home visits to create channels for risk mitigation. The synergy between these two approaches is the key to balancing efficiency and ethical rights.

### 4.3 Synergistic effects of dual embedding: from bridging tensions to value symbiosis

In the practice of Tianjin's Primary Digital Health Community, process embedding and relational embedding have formed a highly efficient synergy, providing a practical pathway to bridge the tension between “precision and warmth” and achieve value symbiosis. On the one hand, process embedding frees up resources and space for relational embedding through precision and efficiency. On the other hand, relational embedding builds trust between doctors and patients, enhances the authenticity and completeness of patient health data, and in turn improves the accuracy of AI-assisted diagnosis, thereby strengthening the operational foundation of the technology.

Process embedding lowers the barriers to care through service convenience, forming a “hard foundation”; relational embedding enhances patient retention by rebuilding trust, injecting “soft power.” Taking diabetes management as an example, the 23.6% decrease in average monthly medical expenses per patient is precisely the result of the synergistic effects of this dual embedding. Process embedding achieves precise cost control at the institutional level, while relational embedding stimulates proactive health management at the individual level; together, they achieve the dual goals of cost control and health improvement. Ultimately, the synergistic interaction of these two approaches enables the digital health community to overcome the challenges of a lack of warmth and imprecision, establishing a virtuous cycle of improved efficiency and service acceptance.

Although the “Process-Relational” dual embedding mechanism has demonstrated significant overall effectiveness and can serve as a model of best practices for digital transformation across regions, a cross-verification of official documents at multiple levels reveals that structural tensions remain that are difficult to fully resolve during the implementation phase. The ethical contradictions mentioned in the preceding ethical analysis, such as algorithmic bias and the digital divide among the older adults have translated into four types of practical implementation barriers. First, while process embedding brings efficiency gains through standardization, digital tasks such as system data entry and online follow-ups have increased the administrative burden on frontline medical staff. Second, the online service system creates digital barriers for the older adults, those with low educational attainment, and patients with chronic conditions who lack self-care abilities. Third, AI-assisted diagnosis and intelligent prescription review algorithms, which are trained solely on data from common local diseases, carry potential algorithmic fairness biases. Fourth, there is an uneven distribution of digital equipment and cloud pharmacy resources across urban and rural areas, with primary healthcare facilities in remote suburbs receiving fewer resources than central urban sites.

In response to the above issues, Tianjin has simultaneously leveraged relationship-based integration to establish safety-net services, such as in-home nursing, in-person service counters, and offline communities for chronic disease management to mitigate these challenges, thereby alleviating the conflict to some extent. However, such endogenous conflicts cannot be completely eradicated by digital platforms, which means that when other regions adopt this model and promote digital reforms, they must design supporting resolution mechanisms in advance; otherwise, they are highly likely to face similar governance challenges.

## 5 Key programmatic elements

During the initial promotion phase of Tianjin's Primary Digital Health Community, tensions emerged including physicians' adaptation challenges to standardized HIS systems and the high barriers posed by online digital technologies for older adults populations. The dual embedding “process-relational” approach not only resolved the operational dilemma of balancing precision with human touch but also drove a triple-tiered value leap in grassroots healthcare service delivery models at the macro level. The success of Tianjin's Primary Digital Health Community lies in empowering physicians and supporting patients to achieve synergistic precision and warmth, transforming tensions into momentum for deepening governance reforms.

### 5.1 Shifting from extensive supply to targeted precision delivery

Traditional primary healthcare services typically operate under a one-size-fits-all model of extensive provision, resulting in a significant disconnect between resources and actual needs (26). Digital platforms enable the profiling of service demands through the collection and analysis of massive datasets. For instance, Tianjin's Primary Digital Health Community can precisely identify key populations such as those with diabetes and hypertension for refined management, directing limited public resources to the most needy individuals and areas. This shift from a flood-irrigation approach to precision-targeted drip irrigation signifies a fundamental leap in resource allocation efficiency and health outcomes for primary care services. The success of this model also provides crucial lessons for future digital technology applications in other public service sectors like older adults care and social security.

### 5.2 Shifting from one-way management to two-way interaction

Under the traditional primary healthcare model, the relationship between doctors and patients was largely a one-way relationship between a manager and those being managed. Digital platforms empower frontline workers and connect diverse stakeholders, creating rich channels for communication and interaction. Residents are no longer merely passive recipients of services; they can use the platform to provide feedback on their needs, evaluate services, participate in decision-making, and form mutual support networks among patients. Patients interacting with doctors via mobile apps and taking an active role in their own health management exemplify this transformation. This shift from one-way management to two-way interaction reshapes the relationships between doctors and patients as well as between government and society. It enhances participation and coordination in primary healthcare governance and represents a crucial step toward a governance model characterized by “joint construction, co-governance, and shared benefits.”

### 5.3 From the dominance of instrumental rationality to the return and integration of value rationality

Digital empowerment dominated by instrumental rationality tends to fall into a narrow logic that prioritizes efficiency above all else. An excessive focus on process optimization and technological application can easily lead to the neglect of humanistic care and fairness, resulting in the erosion of public value (27). The core significance of dual embedding lies in breaking the one-sided dominance of instrumental rationality and shifting digital empowerment from an efficiency-first approach to a value-led one. The foundation of efficiency established through process embedding is no longer the ultimate goal, but rather serves the pursuit of higher-level values; while the human-centered orientation and value principles embodied by relational embedding establish the value boundaries and developmental direction for technological applications. The practice of Tianjin's Primary Digital Health Community demonstrates that the ultimate goal of digital empowerment is not merely the pursuit of high efficiency and low costs, but the realization of the value pursuit of “putting people's health at the center.” This marks a profound transition in grassroots healthcare digitization, moving from being dominated by instrumental rationality to a return to and integration with value rationality.

## 6 Discussion/implications and lessons learned

Facing the inherent tension between “precision” and “warmth” in primary healthcare services in the digital age, this paper constructs a Dual embedding analysis framework of “Process-Relational”. Drawing on a case study of Tianjin's Primary Digital Health Community, it reaches the following core conclusions: The key to digital technology empowering primary healthcare lies not in the advanced nature of the technology itself, but in the ability to build an ecosystem that deeply integrates “precision” and “warmth.” The dual embedding platform proposed in this paper achieves precise service delivery and improved efficiency through process embedding, while conveying humanistic care through relational embedding. The synergy between these two approaches effectively resolves the “precision-warmth” paradox, driving a triple leap in the efficiency, governance, and value of primary healthcare services.

Tianjin's experience has fully demonstrated the effectiveness of this model and offers important insights for policymakers: First, it is essential to maintain a balanced approach that combines precision with warmth. While leveraging standardized information systems and intelligent tools to address supply-demand mismatches, we must avoid allowing technology to replace human interaction, and instead use digital platforms as a medium to strengthen the emotional bond between doctors and patients (28). Second, the roles of frontline personnel must be redefined. Practitioners such as family doctors should be transformed from subjects of surveillance into key hubs connecting technology with residents, freeing them from tedious administrative tasks so they can devote themselves to more meaningful, patient-centered care. Finally, the “warmth” of interpersonal relationships should be used to bridge the digital divide, ensuring that the benefits of digitalization reach vulnerable groups such as the older adults, thereby enhancing the equity and resilience of the healthcare system (29). In summary, the key to the success of primary care digitization lies in breaking down the dichotomy between precision and warmth, and designing the system as a unified and interdependent whole. This not only provides a reference that combines theoretical depth with practical feasibility for addressing the challenges of policy design in primary care digital transformation, but also points the way toward a future shift in thinking. It aims to create a dynamic, symbiotic system where technology enhances humanistic care while humanistic care calibrates technology, thereby truly realizing the core objective of “putting people's health at the center.”

Tianjin's Primary Digital Health Community are able to achieve the synergistic operation of process embedding and relational embedding, relying on a comprehensive city-level fiscal safety net and a supporting system of vertical governance, which serve as indispensable prerequisites for the implementation of this model. From a fiscal perspective, Tianjin has established a regular municipal-level special budget for grassroots healthcare digitization. The costs of cloud platform development, bulk procurement of smart devices, annual system upgrades, cloud pharmacy logistics and distribution, and home care subsidies are all uniformly coordinated and allocated by the municipal government. These costs are not passed down to individual community health service centers, thereby preventing grassroots institutions from scaling back online and offline support services due to insufficient funding. At the same time, a long-term mechanism for annual operation and maintenance subsidies has been established to continuously cover expenses related to AI system updates and the online management of chronic diseases, ensuring the sustainable provision of digital services. From an institutional perspective, the city's health system has achieved vertically integrated, centralized management. Standardized renovations of 266 primary healthcare institutions were uniformly completed, and the city-wide procurement and deployment of cloud-based HIS and public health information systems were standardized, thereby eliminating data silos and inconsistencies in operational standards across districts and counties at the top-level; This is complemented by a citywide unified evaluation system for family doctor contracts and a collaborative management mechanism integrating medical and preventive care, providing administrative safeguards for multi-stakeholder coordination and long-term doctor-patient communication. In terms of hardware and human resources, Tianjin has achieved full broadband and mobile network coverage across all urban and rural areas, and has uniformly deployed cloud-based telemedicine kits and smart monitoring terminals for chronic diseases; the city has also maintained stable staffing levels for primary care medical personnel and established dedicated digital operations and maintenance positions. These measures support healthcare professionals in shifting away from repetitive administrative tasks to provide patient centered follow-up services. These four supporting conditions fiscal resources, institutional frameworks, infrastructure, and human resources collectively form the foundational support for the operation of the “Tianjin Model.” They also represent the core barriers preventing most regions with limited fiscal resources and insufficient district and county-level coordination capabilities from fully replicating this model.

From a horizontal comparative perspective in the international context, Tianjin's digital approach characterized by government coordination and integration of public healthcare and disease prevention exhibits fundamental institutional differences from mainstream global models of primary digital healthcare. Previous discussions that merely touched upon the differences in digitalization between China and other countries have had obvious limitations. First, primary healthcare in OECD countries in Europe and the United States is dominated by market-driven private clinics and private outpatient facilities. Digital platforms are primarily developed and operated by commercial technology companies, and there is a lack of a comprehensive public financial safety net mechanism. These systems can only rely on the market to spontaneously optimize process integration, making it difficult to establish a comprehensive doctor-patient collaboration network through administrative measures. Consequently, they inherently lack the institutional foundation for large-scale relationship integration. Second, in developing economies such as Southeast Asia and Africa, public health funding at the primary care level is limited, and digital infrastructure coverage in both urban and rural areas is low. This makes it impossible to establish unified, centralized platforms for cloud-based medical examinations and cloud pharmacies; only basic online appointment booking functions can be implemented, making it difficult to simultaneously achieve end-to-end process standardization and long-term, emotionally engaging services. Third, although Nordic welfare states have ample public finances, primary healthcare falls under the local self-governance system. The lack of vertical coordination authority at the municipal level creates greater obstacles to cross-regional resource allocation and the implementation of unified doctor-patient communication platforms. Overall, the challenge of balancing precision and warmth exists across different healthcare systems. While the dual embedding analysis framework of “Process-Relational” offers foundational theoretical value, Tianjin's comprehensive implementation measures, which rely on strong government coordination, a public healthcare system, and dedicated municipal funding cannot be directly applied to overseas healthcare systems characterized by marketization, decentralized autonomy, and fiscal constraints.

If a dual embedding mechanism like this is to be implemented in areas within the country, such as counties and remote rural regions, where digital infrastructure is underdeveloped and fiscal resources are limited, four fundamental conditions must be met to ensure both efficiency and a human-centered approach. First, establish a unified digital coordination fund at the county level and set up a long-term special fund for operations and maintenance to share the costs of procuring cloud systems and smart devices, thereby preventing individual township health centers from bearing digital expenses alone. Second, improve the county-level integrated health management system by standardizing data formats for primary care and public health, breaking down information barriers between township and village-level medical institutions, and enabling the sharing of resources within the region. Third, provide essential digital hardware for vulnerable groups by permanently maintaining in-person service counters for the older adults, people with disabilities, and those with low educational attainment, while supplementing these with simple smart devices and regular home visit services to proactively bridge the digital divide. Fourth, optimize the allocation of healthcare personnel at the grassroots level, streamline digital reporting and evaluation tasks, and set aside sufficient time for in-person follow-ups and doctor-patient communication. Once these mandatory conditions are met, local authorities may flexibly adjust the scale of AI tool and cloud platform development based on their own fiscal capacity, prioritizing core functions such as basic diagnosis and treatment and chronic disease management, without needing to replicate Tianjin's entire suite of intelligent systems.

This study takes Tianjin's Primary Digital Health Community as case study. Given the unique characteristics of this case in terms of fiscal policy, infrastructure, and coordinated management, the conclusions are distinctly context-dependent and have limited applicability to the market-driven, decentralized healthcare systems of Europe and the United States; they are applicable only to government-led public healthcare settings. Based on the three-tiered analysis presented earlier, Tianjin's local supporting infrastructure, international differences, and implementation conditions in less developed regions within China, the external validity of the study's conclusions can be systematically defined in a hierarchical manner. First, the core theoretical mechanism of “dual embedding” that balances precision and warmth possesses cross-regional and cross-national universality. Regardless of different economic regions within China or diverse public-private healthcare systems overseas, digital transformation at the primary care level will invariably face an inherent tension where technological efficiency crowds out humanistic care; the analytical framework of synergistic coexistence between process embedding and relational embedding can serve as a universal analytical tool. Second, the external validity of Tianjin's localized “Four Clouds” region-wide coordinated implementation plan is relatively low, as it faces three types of rigid contextual constraints. In terms of fiscal constraints, the region-wide cloud platform, AI-assisted diagnosis and treatment, and centralized pharmacies require sustained, substantial public financial investment; in terms of institutional constraints, the model relies heavily on vertical coordination by the municipal health authority and the integrated public healthcare and prevention system; and in terms of demographic and geographic constraints, while high population density in urban areas facilitates collaboration among diverse stakeholders, implementation costs in dispersed rural areas are significantly higher. In summary, while regions both domestically and internationally can draw on the balancing logic of dual embedding, they cannot directly replicate Tianjin's entire development model. Instead, they must reconstruct localized digital solutions tailored to their local fiscal capacity, healthcare governance systems, and population distribution.

## 7 Acknowledgment of any conceptual or methodological constraints

This study has several limitations that warrant clarification. First, the core analytical material for this paper consists of officially released secondary texts. Such materials carry an institutional bias toward promoting performance achievements, making it difficult to fully capture the actual conflicts encountered at the grassroots level and the genuine concerns of vulnerable groups. Although this study employs methods such as cross-verification of multiple sources, identification of implicit governance shortcomings in the texts, and integration of publicly available feedback from both medical professionals and patients to mitigate narrative biases, it still cannot compensate for the in-depth, individualized insights that face-to-face interviews can provide.

Second, it uses Tianjin's grassroots digital health consortium as a representative case study. As a relatively mature practice of digital empowerment in China's urban-rural fringe and suburban areas, it has formed a Dual embedding analysis framework of “Process-Relational.” However, the replicability of this model in rural areas with weaker digital infrastructure, lower population density, and more dispersed service networks remains to be verified. The high level of resource integration and institutional support observed in the Tianjin case may be difficult to replicate directly in rural settings with stronger resource constraints. The applicability of this model requires adjustments based on regional characteristics.

Finally, the study lacks supporting long-term follow-up data. The existing short-term data cannot systematically examine the long-term impact of the dual embedding mechanism on the stability of doctor-patient trust, the dynamic adaptation of vulnerable groups to digital services, and the sustainable operation of primary-care service networks; nor can it capture the potential changes and governance challenges that may arise after the model is implemented over the long term. Future research could adopt a mixed-methods design combining longitudinal tracking data, cross-regional case comparisons, and semi-structured interviews to expand and refine this analytical framework across multiple dimensions, including temporal, geographical, and individual perceptions.

## Data Availability

The original contributions presented in the study are included in the article/supplementary material, further inquiries can be directed to the corresponding author.
